# Impact of Close and Positive Margins in Transoral Laser Microsurgery for Tis–T2 Glottic Cancer

**DOI:** 10.3389/fonc.2017.00245

**Published:** 2017-10-16

**Authors:** Ivana Fiz, Francesco Mazzola, Francesco Fiz, Filippo Marchi, Marta Filauro, Alberto Paderno, Giampiero Parrinello, Cesare Piazza, Giorgio Peretti

**Affiliations:** ^1^Department of Otorhinolaryngology – Head and Neck Surgery, University of Genoa, Genoa, Italy; ^2^Department of Otorhinolaryngology – Head and Neck Surgery, Katharinenhospital, Stuttgart, Germany; ^3^Nuclear Medicine Unit, Department of Radiology, Uni-Klinikum Tuebingen, Tuebingen, Germany; ^4^Department of Internal Medicine, University of Genoa, Genoa, Italy; ^5^Department of Otorhinolaryngology – Head and Neck Surgery, University of Brescia, Brescia, Italy; ^6^Department of Otorhinolaryngology – Head and Neck Surgery, Fondazione IRCCS – National Cancer Institute of Milan, University of Milan, Milan, Italy

**Keywords:** laryngeal cancer, early glottic cancer, transoral laser microsurgery, CO_2_ laser, surgical margins, endoscopy, recurrence-free survival, disease-specific survival

## Abstract

**Introduction:**

Transoral laser microsurgery (TLM) represents one of the most effective treatment strategies for Tis–T2 glottic squamous cell carcinomas (SCC). The prognostic influence of close/positive margins is still debated, and the role of narrow band imaging (NBI) in their intraoperative definition is still to be validated on large cohort of patients. This study analyzed the influence of margin status on recurrence-free survival (RFS) and disease-specific survival (DSS).

**Methods:**

We retrospectively studied 507 cases of pTis–T1b (Group A) and 127 cases of pT2 (Group B) glottic SCC. We identified the following margin status: negative (*n* = 232), close superficial (*n* = 79), close deep (CD) (*n* = 35), positive single superficial (*n* = 146), positive multiple superficial (*n* = 94), and positive deep (*n* = 48) and analyzed their impact on RFS and DSS. Close margins were defined by tumor-margin distance <1 mm. Pre-TLM margins were defined by white light in 323 patients, whereas NBI was employed in 311 patients.

**Results:**

In Group A, DSS and RFS were reduced in positive multiple superficial and positive deep margins (DSS = 96.1 and 97%, both *p* < 0.05; RFS = 72%, *p* < 0.001 and 75.8%, *p* < 0.01). In Group B, DSS was reduced in positive multiple superficial margins (82.4%, *p* < 0.05). RFS was reduced in positive single superficial, positive multiple superficial, and positive deep margins (62.5, 41.2, and 53.3%, *p* < 0.01). In the entire population, RFS was reduced in CD margins (77.1%, *p* < 0.05). Use of NBI led to improvement in RFS and DSS.

**Conclusion:**

The study indicates that close and positive single superficial margins do not affect DSS. By contrast, all types of margin positivity predict the occurrence of relapses, albeit with different likelihood, depending on stage/margin type. CD margins should be considered as a single risk factor. Use of NBI granted better intraoperative margins definition.

## Introduction

Early glottic squamous cell carcinoma (SCC) has an excellent local control rate (LCR) independently of the treatment choice. In particular, for T1 category, LCR is reported to range from 86 to 95% for transoral laser microsurgery (TLM) and from 85 to 94% for radiotherapy (RT) (1–4). In T2 lesions, RT shows a slightly worse LCR, ranging from 67 to 76%, with respect to TLM (76–89%) (1–8). However, a large meta-analysis of 11 studies, involving 1,135 patients, failed to identify any difference in LCR of patients with early glottic SCC receiving TLM or RT, even though overall costs were lower with TLM (7). Despite the above reported similar LCR, present evidence suggests that RT may perform worse than TLM in terms of disease-free survival (9); moreover, the radiation therapy approach seems to entail a higher risk for subsequent total laryngectomy in comparison with TLM (10).

On the other hand, the choice of TLM implies the assessment of a series of patient-dependent and technique-related factors, to obtain the most favorable therapeutic outcome (11–14). One of the most influencing factors in TLM is definitively represented by surgical margins status after surgery. This issue is complicated by the lack of a homogeneous definition of negative, close, and positive margins. In fact, most authors define a margin as “negative” when the tumor-margin distance is >1 mm, “close” when the distance is <1 mm, and “positive” in presence of tumor at the surgical edge. However, management of patients with close and positive margins is still controversial (15–18). Specific indications for surgical re-treatment (by TLM or open-neck approaches) or complementary RT and the impact of such adjuvant treatments on recurrence-free survival (RFS) and disease-specific survival (DSS) are not yet clearly defined.

In light of this, we retrospectively analyzed a large homogeneous cohort of patients affected by early glottic SCC treated by TLM, focusing our attention on the impact of close and positive surgical margins on RFS and DSS, and on the possibility to reduce their rates by using new biologic endoscopy tools such as narrow band imaging (NBI).

## Materials and Methods

We retrospectively analyzed data from a series of 634 untreated patients (560 males, 74 females; mean age, 64.1 ± 10.4 years; age range, 30–88) affected by Tis–T2 glottic SCC who underwent TLM from January 2000 to March 2014 at the Departments of Otorhinolaryngology—Head and Neck Surgery, University of Genoa and Brescia, Italy (Table 1). All patients signed a written informed consent, which was reviewed and approved by the local Ethics Committees and including the use of anonymized patient data for research purposes.

**Table 1:** Demographic characteristics, patients’ stratification by pTNM, margin status, and types of cordectomy performed.

Variables	Entire cohort	Group A	Group B
Number of patients	634	507	127
Age	64.1 ± 10.4	64.1 ± 10.2	64.3 ± 11.2
Male/female	560/74	467/40	93/34
**T category**
pTis	102	102	–
pT1a	316	316	–
pT1b	89	89	–
pT2	127	–	127
**Margin status, no. (%)**
NEG	231 (36.4)	199 (39.2)	32 (25.2)
CS	79 (12.4)	58 (11.4)	21 (16.5)
CD	35 (5.5)	25 (4.9)	10 (7.9)
SS	146 (23)	114 (22.5)	32 (25.2)
MS	94 (14.8)	77 (15.2)	14 (13.4)
DEEP	48 (7.5)	33 (6.5)	15 (11.8)
**Type of cordectomy**
I	48	48	–
II	275	262	–
III	122	111	–
IV	40	27	23
V	141	56	99
VI	8	3	5

*NEG, negative margin; CS, close superficial; CD, close deep; SS, positive single superficial; MS, positive multiple superficial; DEEP, positive deep margins*.

The tumors were intraoperatively assessed by both 0° and 70° rigid telescopes (Olympus Medical System Corporation, Tokyo, Japan and Karl Storz, Tuttlingen, Germany), increasing the accuracy of neoplastic superficial spreading evaluation (19). In all patients since January 2008, preoperative videolaryngoscopy was combined with high definition television (HDTV)-NBI (Olympus Medical System Corporation, Tokyo, Japan) (20). In selected cases, CT or MR was carried out to evaluate anterior commissure, visceral spaces, and laryngeal framework involvement.

Adequate laryngeal exposure in microlaryngoscopy was obtained by different laryngoscopes comprising Sataloff (Microfrance^®^iXomed, Saint Aubin Le Monial, France), Dedo, and Dedo-Ossoff (Pilling, Philadelphia, PA, USA). The lasers used were the Sharplan 1055 S (Sharplan, Tel Aviv, Israel) from 2000 to 2004, and the UltraPulse/Surgitouch CO_2_ laser (Lumenis, Yokneam, Israel) from 2004 to 2014.

Patients were treated by six different types of cordectomies according to the European Laryngological Society classification (Table 1), using “en bloc” or “multi bloc” approaches, in relation to the tumor site, size, category, depth of infiltration, and laryngeal exposure (21–23). Frozen sections were not routinely performed. Extra-surgical margins, when deemed necessary, were taken from the surgical bed after resection of larger lesions and sent separately for definitive histopathologic examination.

On the basis of histopathological reports, the entire cohort was divided in two groups: Group A included 507 (102 pTis, 316 pT1a, and 89 pT1b) subjects and Group B 127 (all pT2) patients (Table 1), staged according to the seventh TNM classification by the American Joint Committee on Cancer (17).

We defined surgical margins as follows: negative (distance tumor-margin >1 mm), close (distance tumor-margin between 0 and 1 mm), and positive (presence of at least carcinoma *in situ* at the surgical margin). In case of extra-surgical margins taken at the end of procedure, these were considered as the definitive surgical margins.

Median follow-up for the entire cohort of patients was 60 months (range, 12–176): those staged as pTis–pT1b with negative margins were followed by videolaryngoscopy every 2 months in the first year, every 3 months in the second, every 4 months in the third, every 6 months in the fourth, and once a year afterward. In patients staged as pT2, MR or CT was added every 6 months for the first 2 years of follow-up even if the endoscopy was negative.

In case of close margins and/or single positive superficial margin, a monthly follow-up and, where indicated, periodic imaging were performed. In case of positive multiple superficial or deep margins, intraoperative recording was reviewed and, after multidisciplinary discussion, an adjuvant treatment (transoral re-resection, open partial laryngectomy, or adjuvant RT) was proposed to the patient. Whenever this option was refused by the patient or deemed too risky for his/her general conditions, a strict watch-and-see policy was followed.

### Statistical Analysis

The SPSS program (SPSS, v. 21.0, IBM, Armonk, NY, USA) was used for statistical analysis. Five-year survival curves were plotted using the Kaplan–Meier method; pairwise over strata log-rank test was used to detect survival differences between groups. Analysis was first performed for the entire patient population and then separately for Groups A and B.

The entry point was the date of laser cordectomy. Differences between survival curves were assessed using log-rank test for margin status variables. Six different margin statuses were considered for the Kaplan–Meier survival curve: negative (NEG), close superficial (CS), close deep (CD), positive single superficial (SS), positive multiple superficial (MS), and positive deep margins (DEEP).

The first studied outcome was DSS, with the end point being patient’s death or last follow-up (censored observations, shown as “+” symbol along the survival line). Patients who died of unrelated causes were excluded from the analysis. The second outcome was RFS, with the end point set at the date of recurrence or at the last available visit (censored observations). Organ preservation (OP) was the third measured outcome, with the end point set at the date of total laryngectomy or at last follow-up (censored observations).

Influence of the routine use of HDTV-NBI in defining superficial resection margins and its impact on RFS, DSS, and OP were also investigated by comparing recurrence rates, disease-specific lethal events, and need for total laryngectomy between patients treated before NBI implementation (pre-NBI group, 2000–2007) vs. those operated on thereafter (NBI group, 2008–2014). This analysis was carried out for the entire group of patients, and for Group A, Group B, and pT1a lesions separately. Differences in number of events between the pre-NBI and NBI groups were assessed using chi-squared or Fisher’s exact tests, as appropriate.

Moreover, relative risk of relapse related to margin positivity, age, tumor stage, use of HDTV-NBI for margin definition, and additional treatment after the intervention was tested by using a Cox multivariate model with backward logistic regression. For T2 patients, influence of tumor pattern of spread (i.e., transcommissural, supraglottic, and subglottic extension, as well as muscle infiltration) was further tested with the same model.

## Results

Among all patients, 288 (45.4%) had positive margins. In particular, 146 (23%) had positive single superficial margins, 94 (14.8%) positive multiple superficial margins, and 48 (7.5%) positive deep margins. One hundred fourteen patients (18%) had close margins, among which 79 (12.5%) CS and 35 (5.5%) CD margins (Table 1).

### Impact of Margin Status on DSS

**Figure 1:**
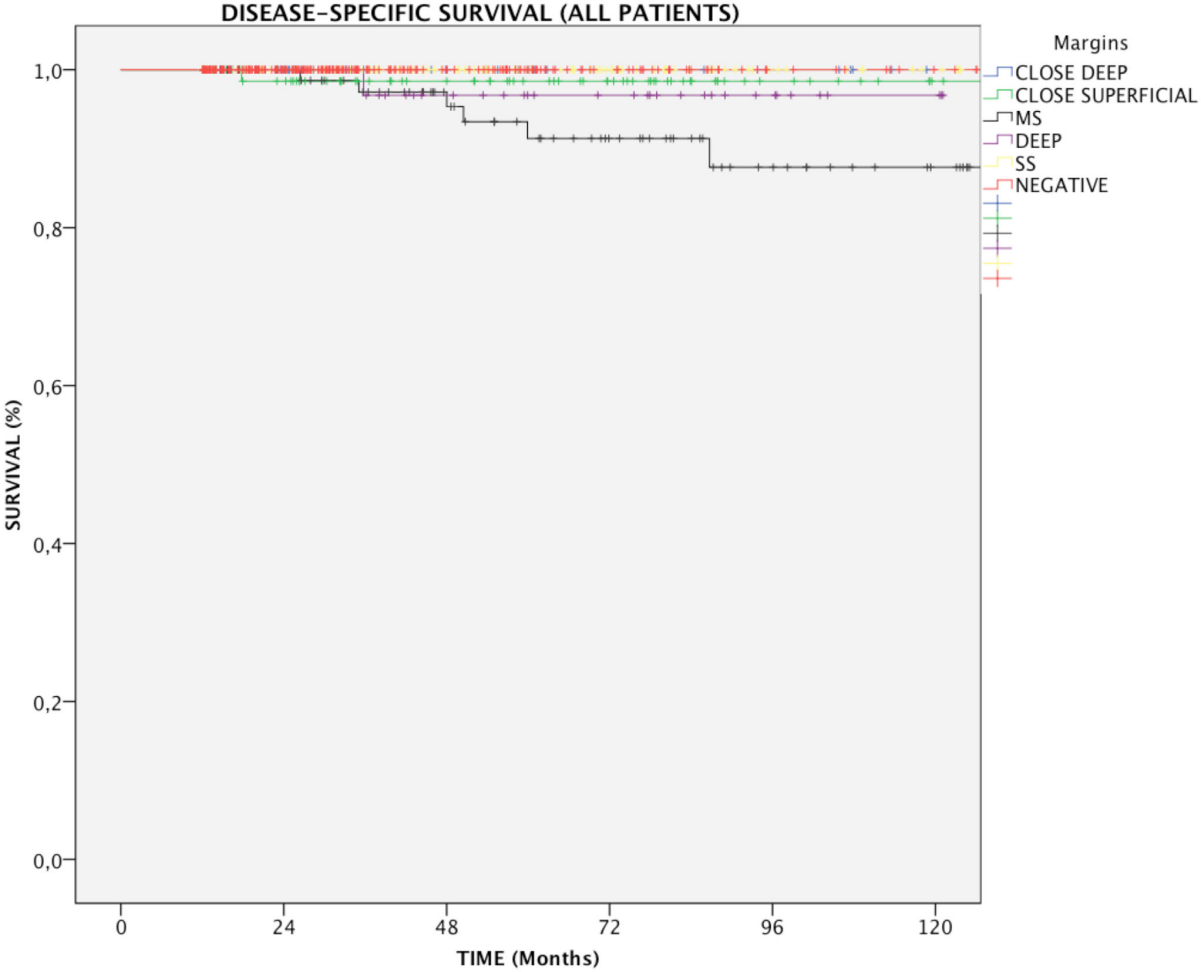
Kaplan–Meier curves for the entire patients cohort showing disease-specific survival in relation to margin status. MS, positive multiple superficial margins; SS, positive single superficial margin; “+” symbol, censored observations.

**Figure 2:**
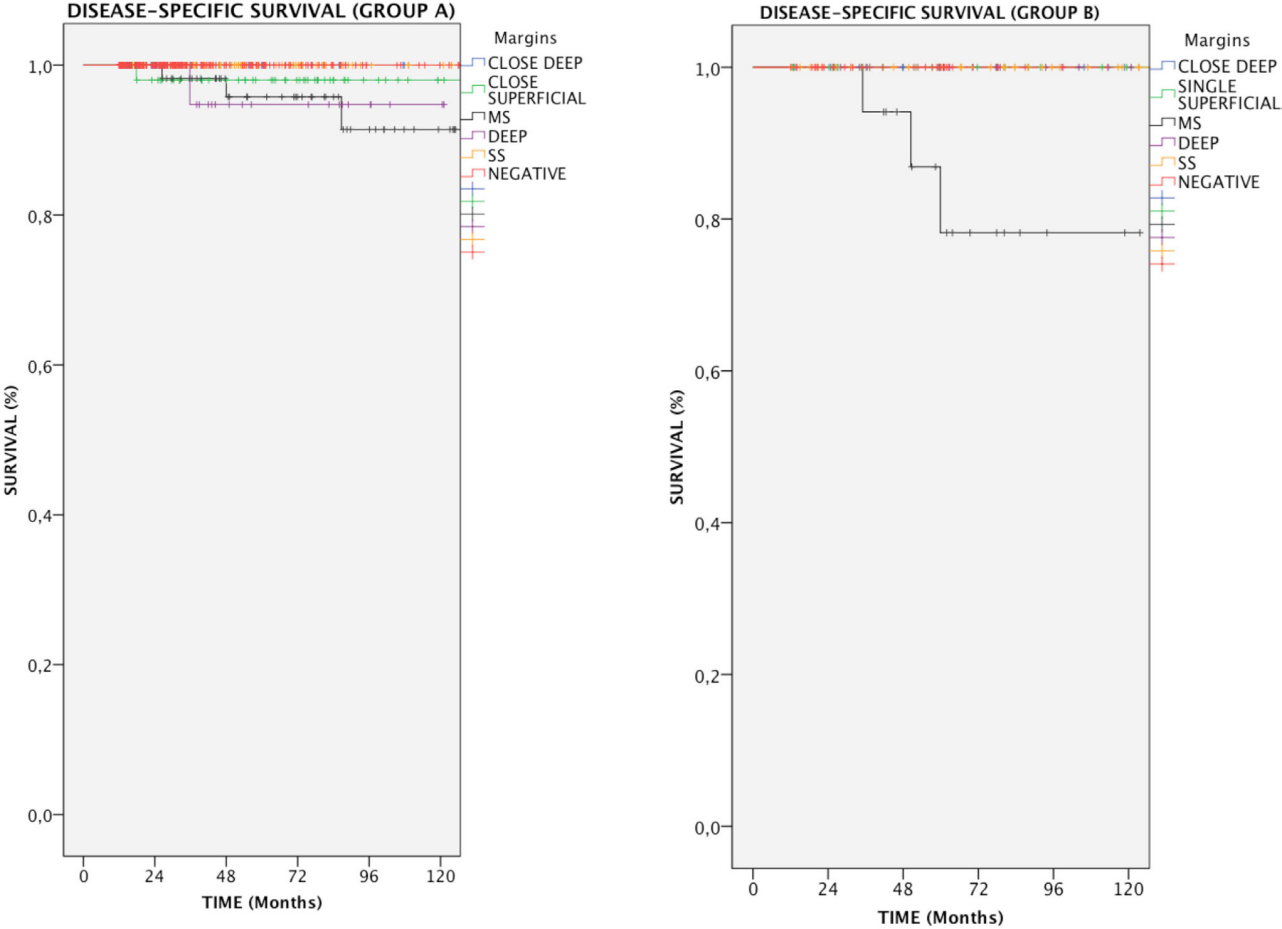
Kaplan–Meier curves showing disease-specific survival in relation to margin status for each group of patients. MS, positive multiple superficial margins; SS, positive single superficial margin; “+” symbol, censored observations.

In the entire cohort, patients with multiple positive superficial margins (MS) had reduced DSS compared with those with negative ones (93.6 vs. 100%, *p* = 0.005, Figure 1). In details, in Group A, both positive multiple superficial (MS) and positive deep margins (DEEP) status were related to a slight, yet significant, reduction in DSS (96.1 and 97%, respectively vs. 100%, *p* < 0.05, Figure 2), whereas in Group B all events were observed in patients affected by positive multiple superficial margins (MS) compared with those with negative ones (82.4 vs. 100%, *p* = 0.019, Figure 2).

### Impact of Margin Status on RFS

**Figure 3:**
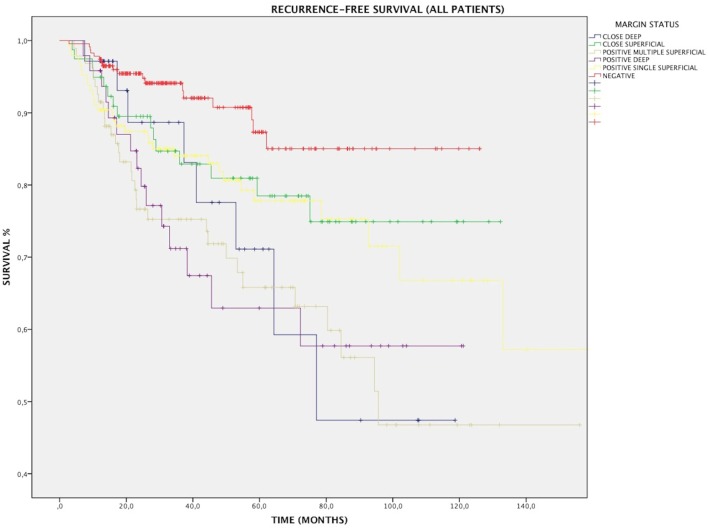
Kaplan–Meier curves for the entire patients cohort showing recurrence-free survival in relation to margin status. MS, positive multiple superficial margins; SS, positive single superficial margin; “+” symbol, censored observations.

Overall, RFS was affected by margin status, as 88.2% of patients with negative and close margins were recurrence free at their last follow-up, compared with 73.3% of patients with positive margins (*p* < 0.001). All subtypes of margins positivity predicted recurrence: RFS for SS, MS, and DEEP margins was 78.8% (*p* < 0.01), 67% (*p* < 0.001), and 68.8% (*p* < 0.001), respectively (Figure 3). At multivariate Cox regression, relative risk of recurrence for this three margin infiltration patterns was 2.1, 3.7, and 3.4, respectively (*p* < 0.01, Table 2).

**Table 2:** Cox regression analysis of factors affecting recurrence-free survival.

	*B*	Sig.	Exp (*B*)	CI 95.0% for exp (*B*)	
				Lower	Upper
**All patients**					
Margins		<0.001	3.703	2.055	6.672
Age		NS	1.002	0.984	1.021
Additional treatment		NS	1.113	0.586	2.113
TNM stage	0.371	0.002	1.853	0.975	3.522
Narrow band imaging	−0.491	0.016	0.612	0.411	0.911
**T2 patients**					
Transcommissural infiltration	0.06	NS	1.062	0.452	2.496
Muscle infiltration	0.667	NS	1.949	0.939	4.043
Hypoglottic infiltration	−0.228	NS	0.796	0.363	1.746
Supraglottic infiltration	−0.071	NS	0.932	0.419	2.071

*B, beta coefficient; Wald, Wald statistic; df, degrees of freedom; Exp (*B*), hazard ratio or relative risk; CI 95.0% for exp (*B*), confidence interval 95.0%; NS, non-significant*.

In Group A, patients with positive margins had a 78.6% RFS, which was reduced in comparison with patients with both negative and close margins together (89.4%, *p* < 0.05). Overall, the difference between negative and all positive margins was significant (*p* = 0.002); in particular, RFS for SS, MS, and DEEP margins was 83.3% (*p* = NS), 72.7% (*p* < 0.001), and 75.8% (*p* < 0.01), respectively. Anterior commissure involvement did not associate with lower RFS (93.5% in tumors reaching the anterior commissure vs. 93.6% in those not involving this subsite, *p* = NS).

**Figure 4:**
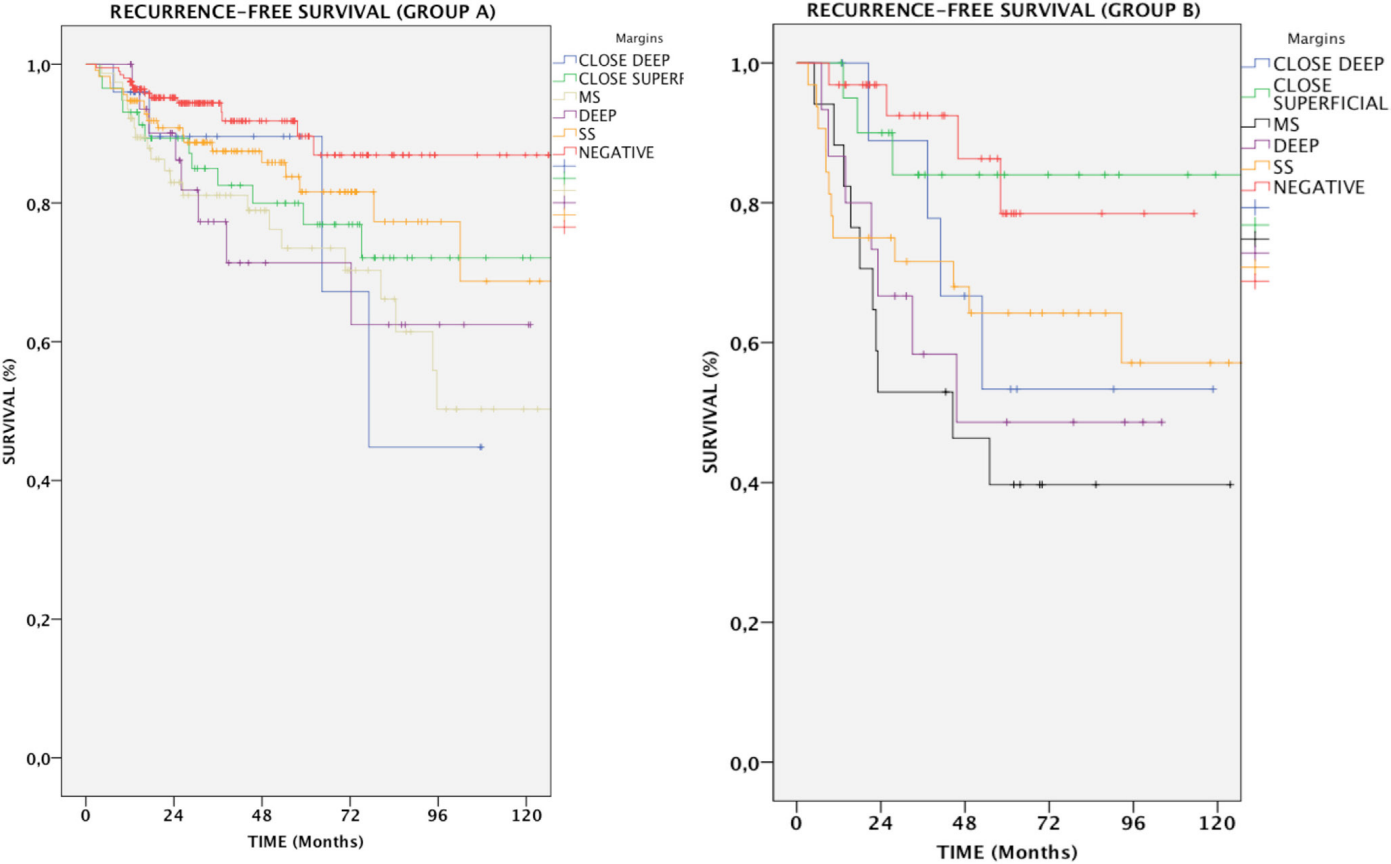
Kaplan–Meier curves showing recurrence-free survival in relation to margin status for each group of patients. MS, positive multiple superficial margins; SS, positive single superficial margin; “+” symbol, censored observations.

In Group B, if patients with negative and close margins were considered as a single group, RFS was 82%. All subtypes of margin positivity were indicative of worse RFS, which decreased to 54.7% in patients whose resection margins were positive (*p* < 0.01). Specifically, the percentage of patients with no evidence of recurrence throughout follow-up was 62.5% (*p* < 0.05), 41.2% (*p* < 0.001), and 53.3% (*p* = 0.012) for SS, MS, and DEEP margin positivity, respectively. Different tumor extension (transcommissural, supraglottic, subglottic, or massive vocal muscle infiltration) did not show any differences in RFS (68.2, 67.8, 70.7, and 74.4% respectively, *p* = NS) (Figure 4).

### Impact of Close Margins

Close margins did not affect DSS. In the entire population, patients with CS margins did not show a significant decrease in RFS (81%, *p* = NS); conversely, the presence of CD margins was related to a significantly increased number of relapses with an RFS of 77.1% and a relative risk of 2.6 (Table 2, *p* < 0.05).

### Organ Preservation

Deep margin infiltration predicted a worse OP in Group A. In fact, no patients with negative margins had to be treated by total laryngectomy, while 6.1% of those with positivity of the deep margin lost the larynx (*p* < 0.01). No significant difference was observed in Group B. Detailed figures regarding RFS, DSS, and OP are shown in Table 3.

**Table 3:** Five-year disease-specific survival (DSS), recurrence-free survival (RFS), and organ preservation (OP) for the entire cohort, and for Groups A and B.

	DSS (%)	RFS (%)	OP (%)
**All patients**			
NEG	100	92.2	98.7
CS	98.7	81	98.7
CD	100	77.1*	91.4*
SS	100	78**	97.3
MS	93.6**	67***	95.7
DEEP	97.9	68***	95.8

**Group A**			
NEG	100	93	100
CS	98.3	79.3*	100
CD	100	84	96**
SS	100	83.3	99.1
MS	96.1*	72***	98.7
DEEP	97*	75.8**	93.9**

**Group B**			
NEG	100	87.5	90.6
CS	100	85.7	95.2
CD	100	60	80
SS	100	62.5*	90.6
MS	82.4*	41.2***	82.4
DEEP	100	53.3*	100

p-Values for comparisons between negative margins and all kinds of positive/close margins are indicated as (*) when <0.05, (**) when <0.01, and (***) when < 0.001.

*NEG, negative margin; CS, close superficial; CD, close deep; SS, positive single superficial; MS, positive multiple superficial; DEEP, positive deep margins*.

### Impact of Additional Treatment

Twelve of 94 patients (12.8%) with positive MS margins underwent additional treatment (11 TLM and 1 adjuvant RT) due to the presence of adjunctive histopathologic risk factors such as high-grade lesions, evidence of perineural spread and/or vascular embolization. The presence of such selection bias could explain why these patients had worse RFS (50%) than those who did not undergo re-treatment (69.5%, *p* = 0.03).

Fifteen of 48 patients (31.2%) with positive deep margins underwent additional treatment (9 TLM and 6 adjuvant RT). Among these, additional treatment showed slight, though non-significant improvement of RFS (73.3%) compared with those who received no further treatment (66.7%, *p* = NS). Detailed figures regarding additional treatments are available in Table 4.

**Table 4:** Impact of additional treatments in all patients on RFS.

	Positive multiple superficial margins		Positive deep margins	
Patients	94	RFS	48	RFS
Without additional treatment	82	69.5%	33	66.7%
With additional treatment	12	50%	15	73.3%
*p*		0.03		NS

*RFS, recurrence-free survival; NS, non-significant*.

### Impact of HDTV-NBI on Margins Evaluation, RFS, DSS, and OP

In all patients, intraoperative use of HDTV-NBI allowed improved evaluation of surgical margins, with a greater proportion of negative margins (50 vs. 30%, *p* < 0.001), reduced number of CS (10 vs. 16%, *p* < 0.001), and multiple superficial margins (15 vs. 17%, *p* = 0.03). Improvement of the rate of negative margins was also observable in Group A (45 vs. 34%, *p* < 0.001) and in pT1a patients (44 vs. 31%, *p* < 0.001). Moreover, use of HDTV-NBI reduced the postoperative findings of CS (8 vs. 15%, *p* < 0.001) and multiple superficial positive margins (13 vs. 18%, *p* = 0.01) in both Group A and pT1a patients (9 vs. 18%, *p* < 0.001; 10 vs. 17%, *p* < 0.001, respectively).

In Group B, use of NBI significantly improved the relative number of negative margins (46 vs. 15%, *p* < 0.001), while CS (24 vs. 29%, *p* < 0.001) and single superficial positive margins (13 vs. 21%, *p* < 0.001) were significantly reduced. As expected, NBI did not affect the rate of positive or CD margins.

In all patients, use of NBI significantly improved RFS (83.9 vs. 78.9% *p* < 0.05). Such improvement was particularly appreciable in negative (94.1 vs. 89.6%, *p* < 0.01) and multiple superficial margins (75 vs. 61.1%, *p* < 0.001). Relative risk, as calculated by multivariate Cox regression, was reduced in patients treated with the help of HDTV-NBI (0.61, *p* < 0.05) (Table 2).

Similarly, NBI was beneficial in negative and MS margins when Group A (*p* < 0.001) or pT1a lesions (*p* = 0.01) were considered. No difference in RFS was detected in Group B. Use of HDTV-NBI also improved DSS in patients with MS margins (Table 5).

**Table 5:** Impact of narrow band imaging (NBI) on 5-year recurrence-free survival (RFS) and disease-specific survival (DSS) for the entire cohort of patients, for Groups A and B, and for pT1a patients.

	Patients (%)		RFS (%)		DSS (%)	
	Pre-NBI	NBI	Pre-NBI	NBI	Pre-NBI	NBI
All patients	323	311	78.9	83.9*	98.8	98.7
NEG	96 (30)	136 (50)***	89.6	94.1**	100	100
CS	53 (16)	26 (10)***	90.6	73.1	100	96.2
SS	78 (24)	68 (25)	80.8	76.5	100	100
MS	54 (17)	40 (15)*	61.1	75***	92.6	95*
Group A (Tis–T1)	251	256	83.3	87.1	99.2	98.8
NEG	85 (34)	115 (45)***	89.4	95.6***	100	100
CS	38 (15)	20 (8)***	89.5	75	100	95
SS	57 (23)	57 (22)	94.3	79	100	100
MS	45 (18)	32 (13)**	66.7	81.2***	95.6	96.9
T1a	160	157	86.3	86.5	99.4	99.4
NEG	50 (31)	69 (44)***	90	97.1***	100	100
CS	29 (18)	14 (9)***	93.1	64.3	100	97.7
SS	36 (23)	36 (23)	86.1	77.8	100	100
MS	27 (17)	15 (10)***	74.1	80**	100	97.6
Group B (T2)	72	55	68.1	69.1	97.2	98.2
NEG	11 (15)	21 (38)***	90.9	85.7	100	100
CS	15 (21)	6 (11)***	93.3	66.7	100	100
SS	21 (29)	11 (20)***	61.9	63.6	100	100
MS	9 (13)	8 (15)	33.3	50	77.8	87.5

p-Values for comparisons between the pre-NBI group of patients and the NBI group in evaluation of margins are indicated as (*) when <0.05, (**) when <0.01, and (***) when <0.001.

*NEG, negative margin; CS, close superficial; CD, close deep; SS, positive single superficial; MS, positive multiple superficial; DEEP, positive deep margins*.

### Impact of Additional Factors on Survival

At multivariate Cox regression analysis, the main factor having an impact on RFS was the surgical margin status (*p* < 0.001) (Table 2). All margin positivity affected RFS; however, in accordance to what demonstrated by the Kaplan–Meier model, multiple superficial and deep margin infiltrations were associated with the highest risk increase (Table 2). Other factors showing a significant incidence on RFS were T category (*p* < 0.01), with T1b and T2 lesions showing increased risk (*p* < 0.05), and use of HDTV-NBI, exerting a protective effect against future recurrences (*p* < 0.05). Age, adjuvant treatment and, for T2 tumors, presence of specific patterns of spread such as transcommissural, supraglottic, subglottic, or massive muscle infiltration, did not affect the risk of developing relapse.

## Discussion

Transoral laser microsurgery is a surgical approach that has emerged as a viable alternative to open-neck approaches and RT as it allows sound oncological results, while preserving organ function and ensuing high salvage rates in case of persistent/recurrent or secondary laryngeal tumors (1–8). Moreover, its favorable cost-effectiveness ratio profile could be helpful in containing ever-growing healthcare costs (7). This type of surgery is mostly applied in early-stage disease and is characterized by a very narrow-margin approach, which makes the process of performing a safe and clean resection challenging. Moreover, the use of laser invariably leads to tissue dehydration and consequent margin shrinkage, which further reduces the ability to obtain widely negative surgical margins at histopathological examination (23). These two aspects force the surgeon to put significant effort in balancing between the best oncological and functional outcomes in each patient.

Actually, one of the most important prognostic factors in TLM remains the status of surgical margins (11, 14, 16). In the present series, we confirmed the feasibility and oncological soundness of this technique, as demonstrated by the high RFS and DSS rates, which favorably compare to those reported in the literature (1, 5, 6). Moreover, it should always be kept in mind that, in TLM for early glottic SCC, RFS does not necessarily have a significant impact on DSS, LCR, or OP, due to the high percentage of recurrences that are salvageable by further conservative treatment options (1). In fact, our data show that even when surgery is executed in the best-case scenario and the surgical margins are completely clean, recurrence is still possible and may affect up to one-tenth of all patients during follow-up. This figure shows a slight variation according to tumor category, which is, however, of minor significance. Use of HDTV-NBI further improved this outcome, as only one in 20 patients with negative margins suffered from disease recurrence after introduction of this bio-endoscopic tool in our routine practice. No patient with negative margins died of disease, thus confirming their good prognostic role.

The same line of thinking can be applied to close surgical margins, as no such patient died of disease. However, the surgeon may expect a higher recurrence rate with respect to fully negative borders, especially in the presence of CD margins, which significantly affected the recurrence rate in our series.

Single superficial margin infiltration had an overall impact on RFS, which was not significantly divergent from CS margins; it has to be noted, however, that, in the setting of T2, risk of recurrence tends to be significant, as 4 in 10 patients were found with recurrence during the course of their follow-up. Again, surveillance in these patients allowed thwarting any disease-related death and DSS was not statistically different from negative and close margins. Moreover, use of HDTV-NBI was especially beneficial in T2 patients, reducing the number of single superficial margin positivity.

It may be surmised that, even though CS and single superficial margins appear to be hallmarks of imperfect disease control, strict follow-up may be sufficient in avoiding the most severe outcomes. Further prospective studies could be aimed to test the validity of such approach against the routine application of adjuvant RT and/or repeated TLM. Our data, however, confirm that CD margins do have an impact on RFS and should not be overlooked.

Actually, even though fatal outcome is a rare occurrence in early glottic SCC (1), the vast majority of events clustered in patients with multiple superficial positive margins. This subpopulation, along with patients with deep margin infiltration, was also burdened by the highest recurrence rate. Moreover, in case of deep margin infiltration, we observed an increased need for total laryngectomy. Our results do not indicate a significant improvement in RFS with the application of adjuvant treatment. However, this analysis was limited by the reduced sample size and by patient selection bias; therefore, to draw any definite conclusions, large controlled prospective trials are needed.

This study has several limitations. First, it is retrospective in nature, analyzing pathologic data gathered at two different institutions over a relatively long time period. Moreover, follow-up was variable, as some patients, treated in the early 2000s, stopped attending visits after a long remission period, while others were recruited at relatively recent dates.

Overall, this study offers a possible key of interpretation of margin results in the framework of clinical decision during long-term follow-up. In particular, it suggests caution in presence of positivity of deep or multiple superficial margins, as these findings are hallmarks of possible disease persistence, especially in higher T categories. Patients with close margins, however, as well as those with single superficial margins, could undergo rigorous follow-up, as their recurrence rate is slightly higher.

## Conclusion

Our data confirm that TLM for early-stage glottic SCC offers sound results in terms of DSS and RFS, as well as a high rate of OP. Survival-related parameters can be effectively stratified by margin status, even though larger studies are needed to definitively assess the opportunity, type, and outcomes of additional treatments in case of positive margins.

## Consent for Publication

The manuscript does not contain any individual person’s data.

## Availability of Data and Materials

The datasets analyzed during the current study are available from the corresponding author on reasonable request.

## Ethics Statement

All patients signed a written informed consent, which was reviewed and approved by the local Ethics Committees and including the use of anonymized patient data for research purposes.

## Author Contributions

IF designed the study; FrancescoM, IF, FilippoM, MF, and AP collected and organized the clinical data. FF performed the statistical analysis. IF, FF, GiampieroP, CP, and GiorgioP drafted and reviewed the manuscript. All the authors read and approved the final manuscript.

## Conflict of Interest Statement

The authors declare that the research was conducted in the absence of any commercial or financial relationships that could be construed as a potential conflict of interest.
